# Supplementary material for: Diversity, composition, altitude, and seasonality of high-altitude windborne migrating mosquitoes in the Sahel: Implications for disease transmission
Source: Front Epidemiol. 2022 Oct 13;2:1001782. doi: 10.3389/fepid.2022.1001782 (PMC10910920; doi:10.3389/fepid.2022.1001782)
Supplement: Supplementary file 1 [file Data_Sheet_1.pdf]

Supplementary Materials:

Diversity, composition, altitude, and seasonality of high-altitude windborne migrating mosquitoes in the Sahel: Implications for disease transmission

By Yaro et al. *** Correspondence: Tovi Lehmann,** tlehmann@nih.gov

Supplementary Results and Discussion

Because no mosquito was collected on 508 control panels that were raised to 40-100 m agl and immediately retrieved during launch and retrieval of the standard panels, these mosquitoes most probably were captured at altitude rather than near the ground (Huestis et al 2019, Florio et al. 2020). Mosquitoes are not captured on the control panels not only because of their short duration but also because launch stations were set in open fields away from humans, animals, and shelters and because most mosquito species are nocturnal, while launch and retrieval occurred during the day. As it was found in over 55% of the genera, i.e., six (or seven) of the 11 genera in Mali (Lehmann *et al.*, 2021; Wilkerson *et al.*, 2021): *Culex, Aedes, Anopheles, Mansonia*, *Mimomiya, Lutzia*, and *Eretmapodites* (Table S1), this migration modality is rather common in mosquitoes. Likewise, high proportion of the species in Mali engage in high-altitude migration because depending on whether species sampled by a single specimen are included, our estimate ranges between 31% and 47%. Yet, the actual proportion is expected to be higher because: i) 473 of 2,576 mosquitoes (18.4%) were not assigned to species, and likely include several new species, ii) our aerial sampling was carried out in the Sahel, whereas other ecozones of Mali, which have distinct mosquito fauna have yet to be sampled, and iii) sampling at higher altitudes, e.g., 300-700 m agl, and during stronger winds (precluded given the helium balloons vulnerability to strong winds) would likely increase the number of specimens and the species diversity. The genus *Culex* predominated in the aerial collection both in terms of the number of mosquito specimens and the number of species (Table S1 and Fig. S1). Test of homogeneity among the three largest genera in this fraction revealed that *Culex* has exhibited higher than expected fraction of species in altitude given its total number of species in Mali (45%, P=0.048, $\chi^2_{[df=1]}$= 3.9 , binomial test). *Culex* has also had the largest number of specimens/species (99).

To ensure we do not include accidentally caught mosquitoes, we excluded species that were represented by a single specimen even though most probably are species that are less abundant in high altitude (see

Figure S1. (Supp. Mat.). Mean number of specimen per species across genera and 95% confidence interval for Nspecies/genus > 7. Mean values are shown above bars.

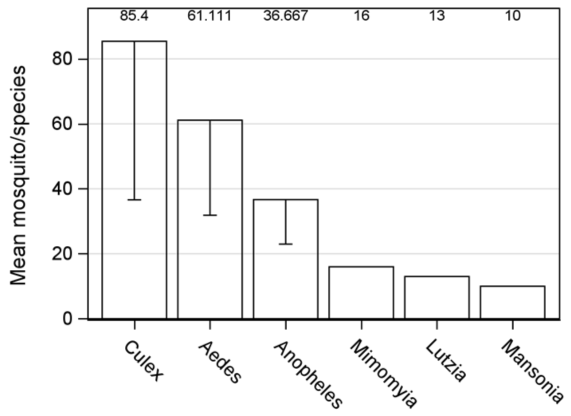

Table S1. Mosquito genera and species in high-altitude in the Sahel and their sample size

Seq.No.	*Genus*	*Species*	No. Specimens
1	*Aedes*	*Ae. fowleri*	238
2	*Aedes*	*Ae. argenteopunctatus*	139
3	*Aedes*	*Ae. quasiunivittatus*	138
4	*Aedes*	*Ae. mali sp. 2*	10
5	*Aedes*	*Ae. mcintoshi*	8
6	*Aedes*	*Ae. mali sp. 1*	7
7	*Aedes*	*Ae. hirsutus*	4
8	*Aedes*	*Ae. circumluteolus*	4
9	*Aedes*	*Ae. vittatus*	2
10	*Aedes*	*Ae. aegypti*[a]	1
11	*Aedes*	*Ae. (Stg.) sp. 1*	1
12	*Aedes*	*Ae. ochraceus*	1
13	*Aedes*	*Ae. triseriatus*	1
14	*Aedes*	*Ae. mali sp. 3*	1
15	*Aedes*	*Ae. mali sp. 4*	1
16	*Aedes*	*Ae. mali sp. 6*	1
17	*Aedes*	*Aedes spp.*	84
18	*Anopheles*	*An. squamosus*	100
19	*Anopheles*	*An. pharoensis*	41
20	*Anopheles*	*An. coustani*	30
21	*Anopheles*	*An. rufipes*	24
22	*Anopheles*	*An. coluzzii*	23
23	*Anopheles*	*An. mali sp. 1*	2
24	*Anopheles*	*An. gambiae*	1
25	*Anopheles*	*An. sp. nr concolor*	1
26	*Anopheles*	*An. mali sp. 2*	1
27	*Anopheles*	*An. cf. coustani 1 NFL-2015*	1
28	*Anopheles*	*Anopheles spp.*	12

**29**	*Culex*	*Cx. perexiguus*	709
**30**	*Culex*	*Cx. cf. watti MAFP5.C5*	308
**31**	*Culex*	*Cx. antennatus*	86
**32**	*Culex*	*Cx. mali sp. 2*	36
**33**	*Culex*	*Cx. MBI-18*	36
**34**	*Culex*	*Cx. watti*	24
**35**	*Culex*	*Cx. nebulosus*	23
**36**	*Culex*	*Cx. bitaeniorhynchus*	18
**37**	*Culex*	*Cx. MBI-03*	18
**38**	*Culex*	*Cx. mali sp. 3*	10
**39**	*Culex*	*Cx. mali sp. 4*	3
**40**	*Culex*	*Cx. duttoni*	3
**41**	*Culex*	*Cx. poicilipes*	3
**42**	*Culex*	*Cx. annulioris*	2
**43**	*Culex*	*Cx. mali sp. 5*	2
**44**	*Culex*	*Cx. cinereus*	1
**45**	*Culex*	*Cx. decens*	1
**46**	*Culex*	*Cx. pipiens*[a]	1
**47**	*Culex*	*Cx. simpsoni*	1
**48**	*Culex*	*Culex spp.*	128
**49**	*Eretmapodites*	*Er. intermedius*	1
**50**	*Lutzia*	*Lu. tigripes*	13
**51**	*Mansonia*	*Ma. uniformis*	10
**52**	*Mimomyia*	*Mi. mimomyiaformis*	16
**53**	*Mimomyia*	*Mi. mediolineata*	1

[a] It cannot entirely be rule out is that using colony specimens as positive controls may have resulted in erroneous identification. Although there is no evidence for this possibility, additional caution is needed when this species is being considered as a high-altitude migrant.

main text). Importantly, two species that appear in our aerial collection as singletons are especially important disease vectors, i.e., *Ae. aegypti*, *Ae. ochraceus*, *An. gambiae*, and *Cx. pipiens* (Table S1) (Braack *et al.*, 2018; Lehmann *et al.*, 2021; Wilkerson *et al.*, 2021). An additional concern, we cannot entirely rule out is that during the early phase of the molecular identification, certain specimens used as positive control might have resulted in possible laboratory error due to contamination. Although there is no evidence for this possibility, the inclusion of *Ae. aegypti, An. gambiae*, and *Cx. pipiens* from our laboratory as positive controls requires additional prudence. The *An. gambiae* specimen (Table S1) was separated and identified by another laboratory as previously described (Huestis *et al.*, 2019) prior to the processing of specimens in our own laboratory, precluding this possibility for that species identification.

Table S2. Female proportion across species (N≥4) sorted by the proportion of females in the aerial collection and the proportion of females exposed to vertebrate blood (see text).

Genus	Species	Females	Female (%)	N (sex)	Exposed	Exposed (%)	N (gono.)
Aedes	Ae. hirsutus	2	50.0	4	ND	ND	1
Lutzia	Lu. tigripes	5	55.6	9	ND	ND	4
Mimomyia	Mi. mimomyiaformis	7	58.3	12	4	100	4
Aedes	Ae. mali sp. 2	5	62.5	8	ND	ND	3
Culex	Cx. mali sp. 2	19	70.4	27	14	93.3	15
Culex	Cx. nebulosus	12	75.0	16	9	100	9
Culex	Cx. bitaeniorhynchus	12	75.0	16	6	85.7	7
Anopheles	An. squamosus	73	76.0	96	46	90.2	51
Culex	Cx. watti	13	76.5	17	8	100	8
Anopheles	An. rufipes	16	80.0	20	12	92.3	13
Culex	Cx. MBI-03	12	80.0	15	4	80.1	5
Culex	Cx. mali sp. 3	8	80.0	10	3	50	6
Culex	Cx. cf. watti MAFP5.C5	210	81.7	257	122	94.6	129
Culex	Cx. antennatus	67	82.7	81	42	93.3	45
Anopheles	An. pharoensis	34	82.9	41	31	100	31
Culex	Cx. perexiguus	512	83.5	613	312	94.3	331
Anopheles	An. coustani	24	88.9	27	18	85.7	21
Culex	Cx. MBI-18	27	90.0	30	14	93.3	15
Aedes	Ae. argenteopunctatus	101	90.2	112	53	88.3	60
Aedes	Ae. quasiunivittatus	104	92.9	112	42	89.4	47
Aedes	Ae. fowleri	205	94.5	217	95	95.9	99
Anopheles	An. coluzzii	21	95.5	22	16	88.9	18
Aedes	Ae. mcintoshi	5	100.0	5	ND	ND	3
Aedes	Ae. mali sp. 1	6	100.0	6	3	60.1	5
Aedes	Ae. circumluteolus	4	100.0	4	ND	ND	2
Mansonia	Ma. uniformis	9	100.0	9	5	100	5

Sensitivity analysis was used to evaluate the effect of the uncertainty (and natural variance) in the relative difference in likelihood of transmission by primary and secondary vectors on our estimates of importance of windborne spread of different pathogens and on the relative roles of different vectors on overall windborne spread of pathogens (see Main Text). Accordingly, we compared the correlations between our best estimates of windborne spread depicted in Figure 4. with those based on high and low values that span the range of the difference between primary and secondary transmission ratios. We considered an infection ratio (approximately equivalent to transmission contribution ratio) of 4:1 as the minimum differential ratio in keeping with the definition of primary and secondary vectors because similar size difference may also be found between two primary vectors in which one contributes 75% and the other 19%, whereas additional three or more secondary vectors contribute less than the remaining 6%. On the other hand, we consider a ratio of 1:0.0025 among the highest differential rate (in their infection rates) because typical sample size per species in most studies ranges between a few hundreds and a few tens of

thousands and the infection rate of the primary vector(s) are near 1% so a ratio of 1:0.0025 implies finding mere 2-3 infected mosquitoes of a sample of 100,000, which very few studies have ever reached not to mention exceeded. Our sensitivity analysis reveals that the estimates of windborne spread based on the mid-range (1:0.1) were highly correlated with those in the extreme low (r=0.99, P<0.0001, N=53) and high of the range (r=0.979, P<0.0001, N=53). When summed over pathogens or mosquito species, high correlations persisted (r>0.95, P<0.0001, N=14, Fig. S1).

[Table S3. Please see separate file in Supplementary Materials.]

Figure S2. Sensitivity of estimates of the relative contribution of windborne transport by different mosquito species (a) and pathogens (b) to the uncertainty in transmission likelihood by primary and secondary vectors (see main text). The relationship between estimates based on the “mid-range” difference between primary and secondary vectors (1:0.1 X-axis), and the lowest difference (1:0.25 Y-axis, top) and highest difference (1:0.0025 Y-axis, bottom) are shown. Values close to the diagonal indicate low sensitivity to different weights or similar patterns across weights. Linear regression between estimators are shown (blue) and observations are labeled by abbreviated vector species and pathogen acronym. Pearson correlation coefficients are shown in top left corner of each panel.

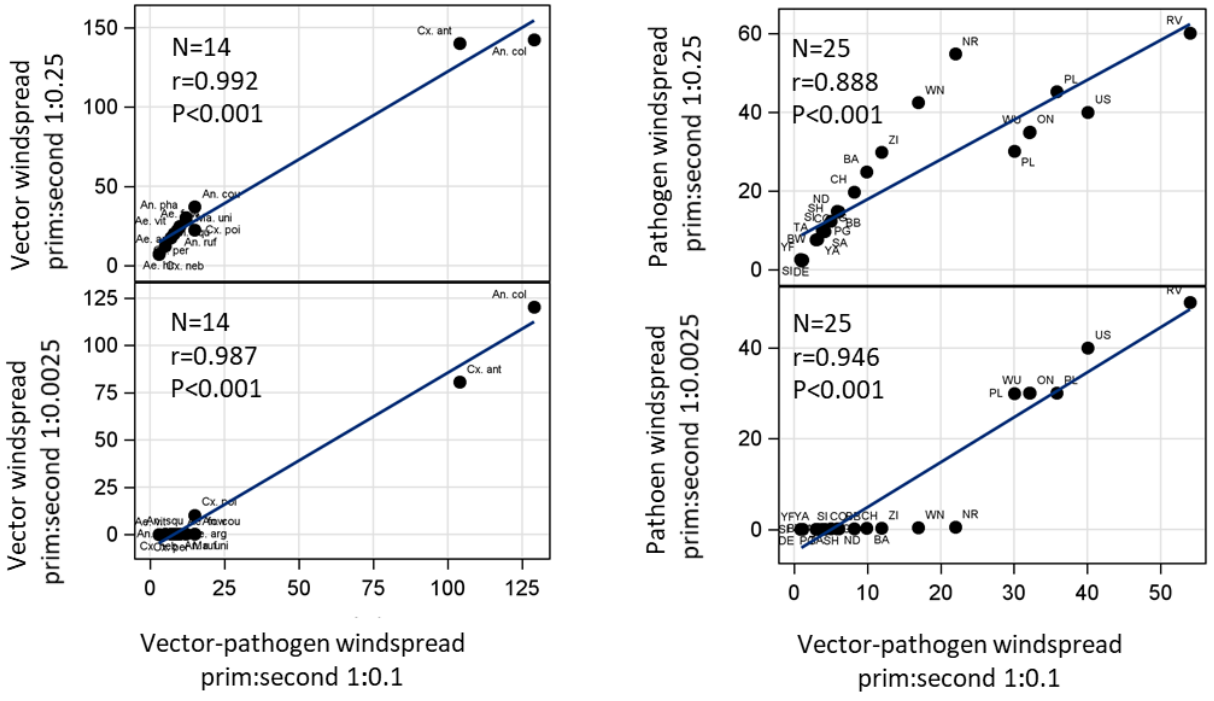

Literature cited

Braack, L., Gouveia De Almeida, A.P., Cornel, A.J., Swanepoel, R. & Jager, C. De. (2018) Mosquito-borne arboviruses of

African origin: Review of key viruses and vectors. *Parasites and Vectors*, **11**, 29.

Huestis, D.L., Dao, A., Diallo, M., Sanogo, Z.L., Samake, D., Yaro, A.S., *et al.* (2019) Windborne long-distance migration of malaria mosquitoes in the Sahel. *Nature*, **574**, 404–408.

Lehmann, T., Kouam, C., Woo, J., Diallo, M., Wilkerson, R. & Linton, Y.-M. (2021) The African mosquito-borne diseasosome: Geographical patterns and range expansion 1. *bioRxiv*.

Wilkerson, R.C., Linton, Y.-M. & Strickman, D. (2021) *Mosquitoes of the World*. Vol. 1 & 2. Johns Hopkins University Press, Baltimore.
